# Transcriptome Assembly, Gene Annotation and Tissue Gene Expression Atlas of the Rainbow Trout

**DOI:** 10.1371/journal.pone.0121778

**Published:** 2015-03-20

**Authors:** Mohamed Salem, Bam Paneru, Rafet Al-Tobasei, Fatima Abdouni, Gary H. Thorgaard, Caird E. Rexroad, Jianbo Yao

**Affiliations:** 1 Department of Biology, Middle Tennessee State University, Murfreesboro, Tennessee, 37132, United States of America; 2 School of Biological Sciences and Center for Reproductive Biology, Washington State University, Pullman, Washington 99164, United States of America; 3 The National Center for Cool and Cold Water Aquaculture, USDA Agricultural Research Service, Leetown, West Virginia 25430, United States of America; 4 Division of Animal and Nutritional Sciences, West Virginia University, Morgantown, West Virginia, 26506, United States of America; Oregon State University, UNITED STATES

## Abstract

Efforts to obtain a comprehensive genome sequence for rainbow trout are ongoing and will be complemented by transcriptome information that will enhance genome assembly and annotation. Previously, transcriptome reference sequences were reported using data from different sources. Although the previous work added a great wealth of sequences, a complete and well-annotated transcriptome is still needed. In addition, gene expression in different tissues was not completely addressed in the previous studies. In this study, non-normalized cDNA libraries were sequenced from 13 different tissues of a single doubled haploid rainbow trout from the same source used for the rainbow trout genome sequence. A total of ~1.167 billion paired-end reads were *de novo* assembled using the Trinity RNA-Seq assembler yielding 474,524 contigs > 500 base-pairs. Of them, 287,593 had homologies to the NCBI non-redundant protein database. The longest contig of each cluster was selected as a reference, yielding 44,990 representative contigs. A total of 4,146 contigs (9.2%), including 710 full-length sequences, did not match any mRNA sequences in the current rainbow trout genome reference. Mapping reads to the reference genome identified an additional 11,843 transcripts not annotated in the genome. A digital gene expression atlas revealed 7,678 housekeeping and 4,021 tissue-specific genes. Expression of about 16,000–32,000 genes (35–71% of the identified genes) accounted for basic and specialized functions of each tissue. White muscle and stomach had the least complex transcriptomes, with high percentages of their total mRNA contributed by a small number of genes. Brain, testis and intestine, in contrast, had complex transcriptomes, with a large numbers of genes involved in their expression patterns. This study provides comprehensive *de novo* transcriptome information that is suitable for functional and comparative genomics studies in rainbow trout, including annotation of the genome.

## Introduction

Rainbow trout (*Oncorhynchus mykiss*), a member of *Salmonidiae* family, is a native species of the Pacific coasts of North America and Russia [1]. They are extensively cultivated worldwide for food, and commercial rainbow trout production significantly contributes to the aquaculture industry in several countries including the USA. In addition, rainbow trout is one of the most extensively studied fish species as it is widely used as a model organism in biomedical research including immunology [2], carcinogenesis [3], physiology [4], nutrition [5], toxicology [6,7], microbial pathogenesis [8], and ecology [9]. More than 9,686 biomedical articles and abstracts have been published on rainbow trout [10].

Over the past decade, international efforts have been made to increase the genomic data on rainbow trout resulting in a significant amount of information in public databases [11–23]. *De novo* transcriptome sequencing has been successfully used for gene discovery, single nucleotide polymorphism (SNP) identification, molecular marker development, detection of expression quantitative trait loci (eQTL), and differential gene expression profiling [24–28]. The available rainbow trout transcriptomic resources include a transcriptome reference sequence that has been developed in our laboratory using a 19X coverage of Sanger and 454-pyrosequencing data [29]. In addition, another reference transcriptome was sequenced in our laboratory representing responses to several stressors affecting the aquaculture production environments [30]. Further, a transcriptome sequence of the anadromous steelhead (*Oncorhynchus mykiss*) was recently reported [31]. While the first study aimed toward assembling a transcriptomic reference for gene discovery, the latter two studies complemented the existing transcriptomic resources and facilitated evaluating gene expression associated with adaptation to ecological and environmental factors in rainbow trout.

Identifying and annotating the coding nucleotide sequences and providing basic functional genomics information will enhance opportunities for genetic improvement of this fish for aquaculture production efficiency and product value and increase its usefulness as a biomedical research model. Recently, unannotated genomic scaffolds and contigs with ~70% coverage of the genome length were assembled from the Swanson River clonal line [32]. More successfully, a draft of the genome sequence has been assembled from a single homozygous doubled haploid YY male from the same clonal line [14]. A gene models approach based on both a genome and transcriptome sequences was used to annotate the genome sequence, predicting 69,676 transcripts. However, the genome sequence still is not complete, with a total length of 2.1 Gb and only 1.023 Gb (48%) of the total assembly anchored to chromosomes [14]. To improve annotation of the under development trout genome sequence and estimate coverage of assembly, a complete and well-annotated transcriptome reference sequence is still needed. Therefore, a *de novo* approach was used in this study to sequence and assemble the rainbow trout transcriptome using in-depth (4,333X) sequence coverage.

Next-generation sequencing is a rapid and cost-effective method for sequencing. However, short sequencing reads generated by most high-throughput sequencing techniques pose difficulties in *de novo* assembly resulting in short/fragmented assemblies of genes [33]. In addition, about 50% of the genes in salmonids are duplicated [34], which makes *de novo* assembly and annotation of the transcriptome difficult and complicates SNP/variant discovery [35–38]. To help overcome these bioinformatics challenges of the trout duplicated genome, we have sequenced the transcriptome of a single doubled haploid fish from a clonal line in an effort to remove sequence variation resulting from polymorphism [14]. This doubled haploid clonal line, which contains two identical copies of each chromosome, was previously established by chromosome set manipulation techniques [39,40] and has been used in sequencing the rainbow trout genome and transcriptome [14,29,41]. Recently, dramatic improvements in genome assembly of *Takifugu rubripes* were achieved by using doubled-haploid individuals compared to the wild types [42].

Housekeeping genes were initially described as genes which are always expressed in the cell [43]. Later, this concept has been refined to refer to genes with constitutive expression that maintain normal cellular functions [44]. In contrast, tissue-specific genes are transcripts whose functions and expressions are favored in specific tissue/cell types [45]. Tissue-specific gene expression is crucial for maintaining specificity and determining complexity of multicellular organisms as they affect the development, function and maintenance of diverse cell types within an organism. Studying the ubiquitous versus the tissue-specific expression of genes enables greater understanding of organismal development, complexity and evolution at the systems level. Large scale gene expression profiling has been done on a small number of organisms [46–51]. In fish, gene expression atlases were characterized in only few model species [52,53]. Identification of housekeeping versus tissue-specific genes provides important molecular information that is needed for genetic improvement of fish for food production and for biomedical research purposes.

Salmonids underwent an evolutionarily recent whole genome duplication event and are in the process of returning to a diploid state [54]. Therefore, some fundamental scientific questions can be explored by decoding the rainbow trout transcriptome including how many genes exist in the rainbow trout, which genes are ubiquitously expressed and which genes and splice variants are uniquely expressed in each tissue to provide tissue specificity. In addition to the fundamental knowledge, this information can be used for the genetic improvement of rainbow trout for aquaculture by eliminating the need to positionally clone genes, facilitating resequencing to identify genetic variants, and identifying candidate genes for traits of interest.

To address the questions above, this study sequenced and *de novo* assembled the rainbow trout transcriptome from 13 vital tissues. High throughput Illumina sequencing in conjunction with the Trinity assembly package were used to: (1) sequence the rainbow trout transcriptome to provide a reference sequence, (2) functionally annotate the transcripts, (3) characterize digital gene expression and alternative splicing in 13 vital tissues; and (4) identify full-length cDNAs in the rainbow trout genome. Illumina sequencing in conjunction with Trinity assembly provided an efficient approach for *de novo* assembly and characterization of the transcriptome with high depth and width of coverage. Results of the *de novo* approach, used in this study, were compared to results of the gene models approach that was previously used in annotating the genome sequence [14].

## Materials and Methods

### Ethics statement

The fish sacrificed for this study was reared and euthanized under protocol #02456 approved by the Washington State University Institutional Animal Care and Use Committee.

### Production of doubled haploid rainbow trout

The rainbow trout from the Swanson clonal line used in the study was produced at the Washington State University (WSU) trout hatchery using previously described techniques [39,40,55,56]. First generation homozygous rainbow trout were produced by androgenesis using gamma irradiation of eggs prior to fertilization [39,40] and by gynogenesis by blockage of first cleavage using hydrostatic pressure shock [39,40,56]. When fish reached sexual maturity, homozygous clones were produced by collecting sperm from homozygous males and doing another cycle of androgenesis, or by stripping the eggs from homozygous androgenetically or gynogenetically produced females and performing gynogenesis by retention of the second polar body [56].

### Tissue collection and RNA isolation

Thirteen different tissues were collected from a single immature (2-year old, 250 g) male homozygous rainbow trout of the Swanson clonal line. Tissues collected were brain, white muscle, red muscle, fat, gill, head kidney, kidney, intestine, skin, spleen, stomach, liver, and testis. Tissues were quick-frozen in liquid nitrogen and were shipped to WVU from WSU in dry ice. Tissues were kept at -80°C until RNA isolation. Total RNA was isolated from each tissue using TRIzol (Invitrogen, Carlsbad, CA) according the manufacturer’s procedure as previously described [29].

### Illumina paired-end sequencing

Construction of RNA-Seq libraries and sequencing on an Illumina Genome Analyzer IIx was performed at Roy J. Carver Biotechnology Center, University of Illinois at Urbana-Champaign. RNA-Seq libraries were constructed with the mRNA Sequencing Sample Preparation Kit (Illumina, San Diego, CA). Briefly, polyA+ messenger RNA was selected from 1 μg of RNA with magnetic oligo (dT) beads, chemically fragmented and converted to cDNA with random hexamers. Double stranded cDNAs were end-repaired, and the 3’-ends were A-tailed followed by ligation of Illumina sequencing and amplification adapters randomly to the ends. The adaptor-ligated cDNAs were loaded onto 2% agarose E-gels (Invitrogen, Carlsbad, CA) and the fraction containing 200–500 bp was excised. Size-selected cDNAs were amplified by PCR with primers that introduced unique barcodes to each library. The final libraries were quantitated with Qubit (Life Technologies, Grand Island, NY) and the average size was determined on an Agilent bioanalyzer DNA7500 DNA chip (Agilent Technologies, Wilmington, DE) and diluted to 10 nM. The 10 nM dilution was further quantitated by qPCR on an ABI 7700. Each library was loaded onto one lane of an 8-lane flowcell for cluster formation and sequenced on an Illumina Genome Analyzer IIx according to the manufacturer’s protocols (Illumina, San Diego, CA). The fastq files were generated with Casava version 1.6.

### Trinity assembly and annotation

All 13 lanes of Illumina paired-end data were used to run Trinity assembler with default parameters. The Trinity software package combines three assembly algorithms: Inchworm, Chrysalis and Butterfly [57]. Assembly algorithms were run in C++ (Inchworm and Chrysalis) and Java (Butterfly) scripts. FASTQ formatted sequencing reads were converted into FASTA format by Fastool software, and extraction and computation of k-mer abundance from the sequencing reads were done by Jellyfish software. During assembly of contigs by Inchworm, minimum k-mer threshold abundance was set to 1 (default). The program was run at default parameters to cluster the Inchworm contigs into components (min_glue <int> = 2, min_iso_ratio <float> = 0.05 and glue_factor <float> = 0.05). Transcript reconstruction from a deBruijn graph by Butterfly was also performed at default parameters (max_number_of_paths_per_node <int> = 10, group_pairs_distance <int> = 500, path_reinforcement_distance <int> = 75, lenient_path_extension = 1). Trinity contigs that were more than 500 nucleotides long were BLAST searched against NCBI non-redundant (NR) protein database. The longest transcript of each Trinity contig group that matched a given protein in the NR database was selected as a representative sequence for each contig group.

### ORF/full-length cDNA prediction and gene ontology analysis

All representative transcripts selected from contigs having hits to the NCBI NR protein database were analyzed by ESTScan [58] to search for an open reading frame (ORF), which distinguishes coding and non-coding sequences [58,59]. Whenever an ORF began and ended within a contig, it was considered as full length. If an ORF began at the first base or ended at the last base, it was not considered as full length. In addition, TransDecoder [http://transdecoder.sf.net] was used to identify ORFs with complete coding sequences. Gene ontology analysis was performed by BLASTx search against the NCBI NR protein database using the Blast2GO suite [60]. Blast2GO analysis provides a controlled vocabulary to describe gene product characteristics in three independent ontologies: biological process, molecular function, and cellular component [61,62].

### Identification of housekeeping and tissue-specific genes

Housekeeping and tissue-specific genes were identified using a CLC genomics workbench. A total of 44,990 transcripts selected as representative sequences for each contig group from all 13 tissues were used as a reference sequence. Reads from each tissue (two libraries from each tissue) were mapped against the reference. Transcripts with RPKM (Reads Per Kilo base per Million) value ≥1 in all tissues were defined as housekeeping genes. For the tissue-specific genes, expression level of a gene in a particular tissue was compared to its expression level in all remaining 12 tissues. For distinction of tissue-specific genes, the fold-change in expression level was set as ≥ 8 fold, i.e. genes with an expression level in one tissue that is equal to 8 fold or higher than the maximum value in any of the other 12 tissues. As explained above, a single doubled haploid individual was used in this study to overcome the assembly bioinformatics challenges of the trout duplicated genome. Therefore, inferences regarding the housekeeping and tissue-specific gene expression should be considered with caution because results may be limited to this fish and to the time period during which the tissues were collected.

### Complexity and composition of tissue specific transcriptome

Sequence reads from each tissue were mapped to the 44,990 transcripts used as a reference sequence in this study. After mapping, numbers of genes expressed in each tissue were reported at four different threshold RPKMs (5, 1, 0.5 and 0.1). Transcripts having an RPKM value above the threshold were counted to obtain the number of genes expressed in each tissue. The mRNA abundance of the tissue-specific genes were calculated by dividing the sum of RPKM values of the tissue-specific genes by the sum of RPKM values of all genes expressed in that particular tissue (at RPKM threshold of 0.5). A similar method of comparing the composition and complexity of tissue-specific transcriptomes was employed by Jongeneel and coworkers [63]. A multivariate Principal Component Analysis (PCA) analysis was applied to cluster tissues types according to gene expression patterns using a CLC genomics workbench.

### Assessment of the assembled rainbow trout transcriptome

Reference proteome sets of seven model fish species with known reference genome (*Danio rerio*, *Oreochromis niloticus*, *Takifugu rubripes*, *Tetraodon nigroviridis*, *Gadus morhua*, *Gasterosteus aculeatus*, and *Oryzias latipes*) were downloaded from the Uniprot database. Rainbow trout protein coding sequences resulting from the Trinity assembly were searched against the reference proteome of each fish species by BLASTx with a cut off E value of 1.00E-10. To obtain the expected range of sequence conservation between model fish species, cDNA sequences of model fish species were downloaded from the NCBI database. The cDNA sequences of each fish species were searched against the reference proteome set of the other model fish species by BLASTx with a cut off E value of 1.00E-10.

### Genome read mapping, annotation and assessment of alternative transcription/splicing

Alternative transcription/splicing events were assessed using the Bowtie2, TopHat and Cufflinks software package [64,65]. First, a rainbow trout draft genome assembly was downloaded from http://www.genoscope.cns.fr/trout-ggb/data/ [14]. Then, sequence reads from all 13 tissues were mapped to the genome reference using Bowtie2/TopHat. Cufflinks was used to generate a transcriptome assembly for each tissue using alignment files from TopHat. Assemblies were then merged together using the Cuffmerge utility. Reads and the merged assembly were then analyzed using Cuffdiff to identify alternative transcripts (produced by alternative splicing/start sites) from each genomic locus (gene).

To identify novel genes, gene loci predicted by Cufflinks were filtered against the trout genome annotated loci first by BLASTn against the mRNAs (E value 10^-5^) then by comparing the genome annotation coordinates (gtf files) using in-house script. TargetIdentifier [66] and TransDecoder [http://transdecoder.sf.net] were used to determine novel genes with ORFs. In addition, an in-house software (available upon request) was used to determine novel genes with 80% and 100% match to the NR database at an E value 10–3.

BLAT [67] with default parameters was applied to map the Trinity transcripts to the reference genome. The pslReps programs in the BLAT suite was used to select the best alignments for each query sequence. BLAT hits were classified based on the percentage of sequence identity covering the reference coding sequence at 100%, 90% and 50% of the entire coding sequence.

## Result and Discussion

### Illumina sequencing and Trinity assembly

To improve assembly and annotation of the rainbow trout reference transcriptome, libraries were constructed from a single double-haploid individual of the Swanson homozygous clonal line that has been used in sequencing the rainbow trout genome [14,41] and in our previous transcriptome assembly [29]. Total RNA was isolated and sequenced from 13 different tissues of vital importance to fish life. These tissues were brain, white muscle, red muscle, fat, gill, head kidney, kidney, intestine, skin, spleen, stomach, liver and testis.

To maximize transcript coverage, cDNA libraries were sequenced on 13 separate lanes of an Illumina’s Genome Analyzer using a paired-end protocol, yielding a total of 1.167 billion paired-end reads (100 bp). The cDNA library and sequencing information is given in Table 1. To allow identification of housekeeping and tissue-specific gene expression, sequences were generated from non-normalized libraries from different tissues. To facilitate the assembly, sequence reads were preprocessed to remove artifacts including sequencing adapters, low complexity reads and near-identical reads to improve read quality and efficiency of assembly [68].

**Table 1:** cDNA library information and summary of the high-throughput sequencing yield.

	Tissue	Number of reads
1	Red Muscle	93,064,168
2	Skin	87,743,778
3	Fat	93,546,068
4	Brain	84,816,430
5	Gill	92,670,670
6	Spleen	93,532,200
7	Head kidney	92,168,818
8	Liver	85,281,910
9	Stomach	91,231,186
10	Intestine	91,613,688
11	Testis	85,389,746
12	White Muscle	86,643,770
13	Kidney	89,642,288

**Fig 1:**
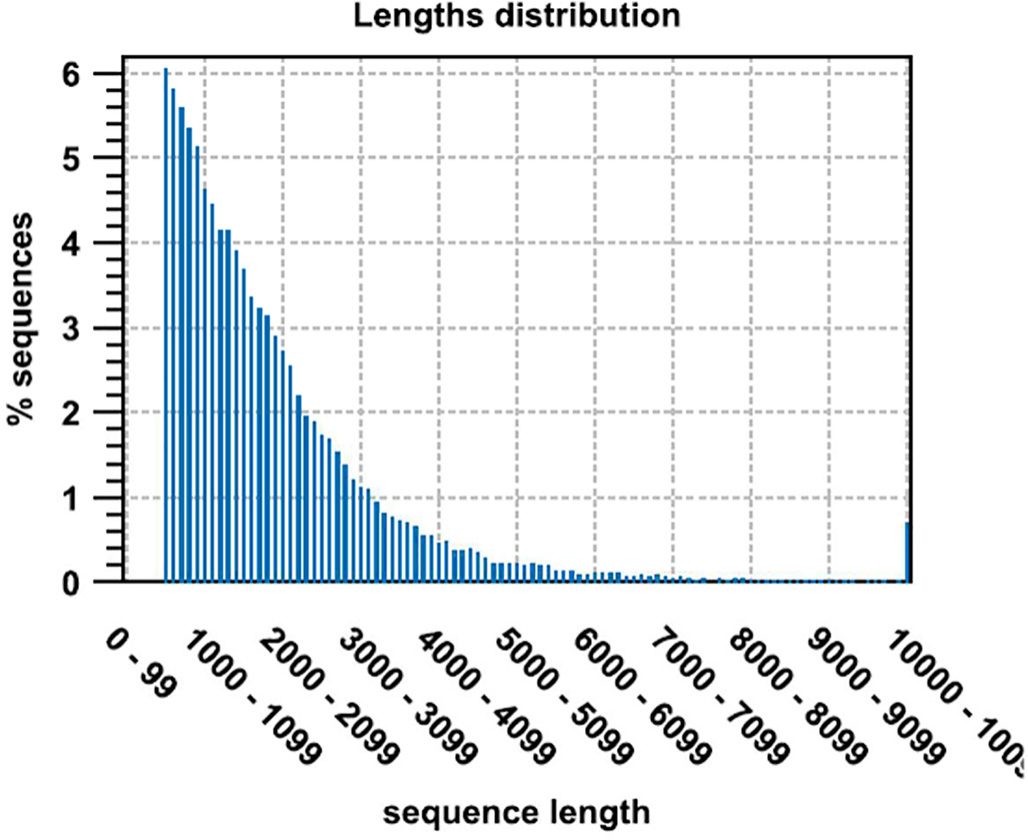
Distribution of contig (≥ 500 nt) length of a rainbow trout Illumina/Trinity transcriptome assembly.

RNA-Seq data were *de novo* assembled using the Trinity assembly package which comprises combining sequence reads into larger contigs (by Inchworm), clustering contigs into a component (by Chrysalis), and producing the most plausible sets of transcripts from these groups (by Butterfly) [57]. An assembly of 1.167 billion paired-end reads gave 1,371,544 Inchworm contigs (contig length > 200bp, ave = 744 bp). Inchworm contigs longer than 500 nucleotides (474,524 contigs) were used for downstream analysis. Assembly statistics and length distribution of contigs are given in Table 2 and Fig. 1. These Inchworm contigs were clustered into a set of connected components to construct deBruijn graphs for assembly components. Each component defines a collection of contigs that are derived from alternative splicing or closely related paralogs [57]. These contigs were categorized into 163,411 components. Of them, 57,467 components contained more than one contig, while the remaining 105,944 were single contig components. The Trinity assembly package was used based on previous studies done in model species that suggest better performance of Trinity over some other assemblers, its ability to construct full-length transcripts, and the quality of the constructed transcripts [57,69].

**Table 2:** Assembly statistics of Illunina paired-end data.

	All contigs	Long contigs (≥ 500 nt)
Number of bases	1,020,368,806	753,301,781
Number of contigs	1,371,544	474,524
N50 (nt)	1,369	2,188
Largest contig length (nt)	54,460	54,460
Smallest contig length (nt)	201	500
Average contig length (nt)	744	1,587

All 474,524 Trinity contigs longer than 500 nucleotides were searched against the NCBI non-redundant (NR) protein database. A total of 287,593 (60.60%) contigs had hits to the database proteins. Importantly, 92.5% (266,188) of these contigs were part of the components with more than one contig, indicating the existence of a large number of transcript variants possibly due to alternative splicing, variable transcription start or termination points, or paralogous loci.

One of the remarkable findings of the project was the failure of a significant number of contigs (39.40% of 474,524 contigs) to have hits to the NR database, a finding similar to that observed previously in rainbow trout [70]. Similarly, in a catfish EST project Wang et al (2010) reported over 40,000 unique catfish sequences containing ORFs had no significant hits to the NCBI protein database [71]. Likewise, three transcriptomes from Antarctic notothenioid fish revealed 38–45% significant BLASTx hits in the NR protein database [72]. The unmatched contigs were used to identify a large number of non-coding RNAs (data will be published elsewhere). In addition, the unmatched contigs may result from mistakes in assembly (contigs from reads with sequence errors) [57], lack of protein sequences of related fish in the database, or “trout-specific” diverged sequences due to the whole genome duplication [73,74].

**Fig 2:**
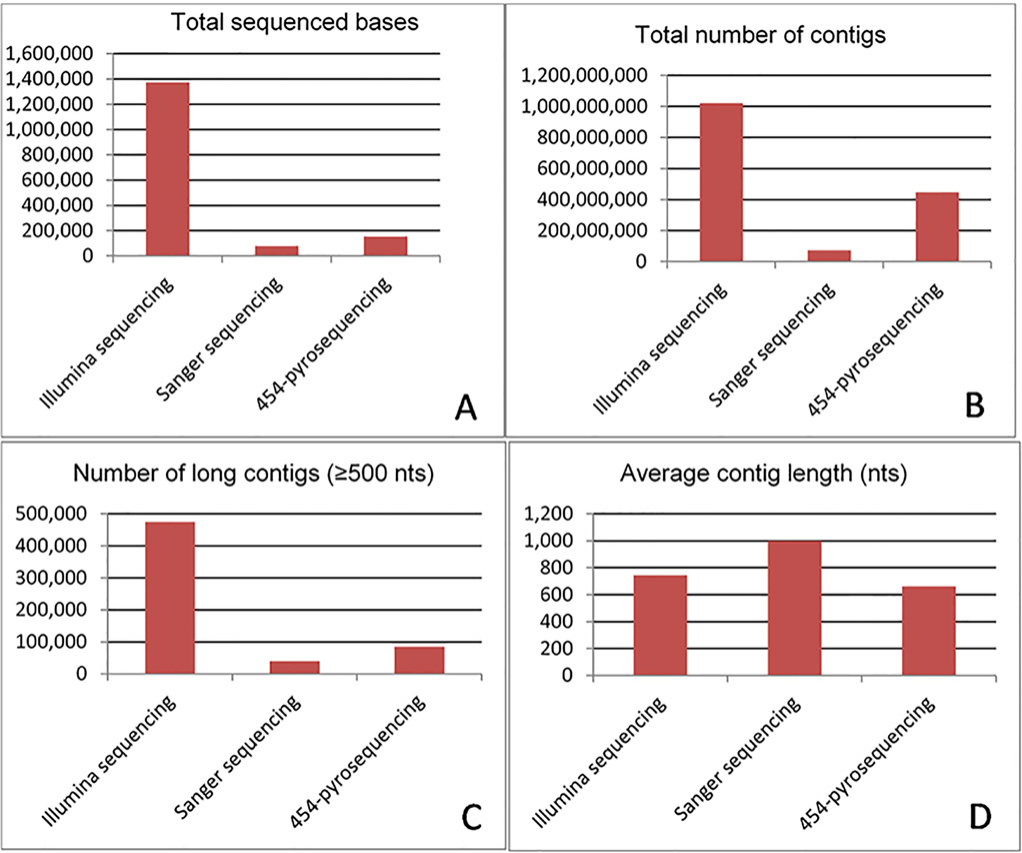
Comparison of total number of sequenced bases (A), total number of contigs (B), number of long contigs (≥ 500 bp) (C), and average length of contigs (D) obtained from Illumina, Sanger-based, and 454-pyrosequencing techniques. Data on Sanger-based and 454-pyrosequencing techniques were obtained from Salem et. al [29].

Previously, we utilized Sanger-based and 454-pyrosequencing approaches for transcriptomic analysis of the rainbow trout [29]. Fig. 2 shows comparisons of the total number of sequenced bases, number of contigs, number of long contigs (≥500 bp), and average length of contigs obtained from Illumina, Sanger-based, and 454-pyrosequencing techniques. Compared to Sanger based and 454-pyrosequencing, Illumina allowed more effective assembly of the transcriptome with tremendous increases in the total number of contigs, total number of long contigs (>500 bp), and average length of contigs. However, the percentage of long contigs (>500 bp) was only 34.59% in the current Illumina/Trinity assembly compared to 56% in the 454-pyrosequencing assembly, which may be attributed to longer sequence reads with454-pyrosequencing (Fig. 2).

### Gene identification and annotation

Transcript annotation was performed by BLASTx similarity search of the Trinity contigs against the NR protein public database. All contigs that had hits to the NR database were further analyzed to select a set of transcripts that could be used for functional genomics downstream analysis and ORF searching. For contigs that belonged to multiple contig components, the longest contig in a component was selected as a reference transcript of each component. For the single contig components, the longest contig was selected when more than one contig had aligned to any database protein with the same gene annotation. After removal of redundant transcripts, 44,990 were selected as a reference set of transcripts, including 34,260 contigs from multiple contig components and 10,730 contigs from single contig components. Of the total 44,990 representative contigs, ESTScan detected 43,824 (97.4%) sequences as having coding regions. The average length and number of the representative contigs is close to those predicted in the rainbow trout genome, 1.97 kb, versus 1.64 kb and 44,990 versus 46,585 in the Trinity assembly and the rainbow trout genome, respectively [14]. In a catfish EST project, a 1.29 kb average length was observed and 98% of the unique sequences with significant hits to a protein database had ORFs [71]. About 2.6% of the contigs in this study (1,166) contained no coding regions (data not shown). These transcripts may represent pseudogenes or transcripts with intron-retaining cDNAs. Most of the contigs having hits to the NR database (97.49%) were identified within coding regions, which supports the credibility of the sequence assemblies.

**Fig 3:**
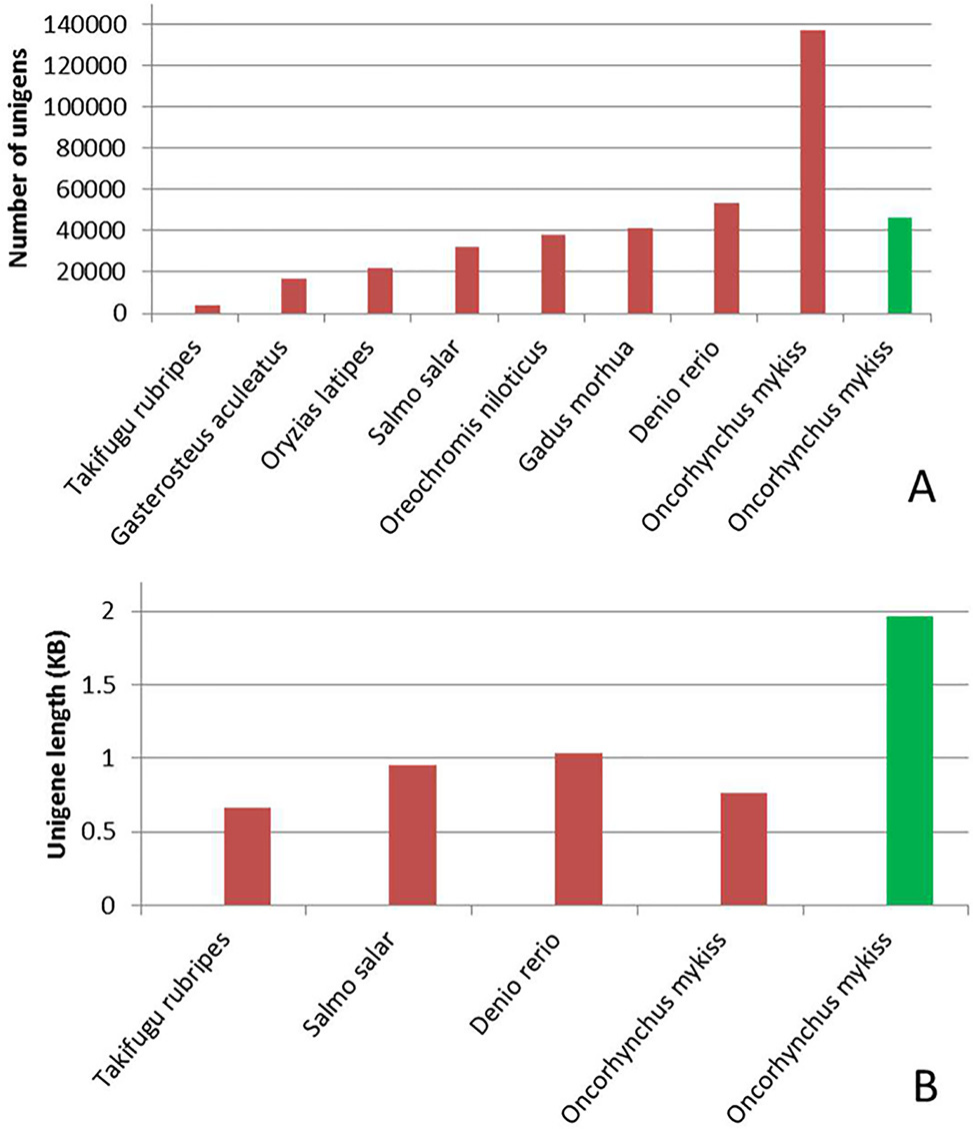
Number of UniGenes of model fish species and rainbow trout UniGenes that are available in the NCBI database (red bars) compared with number of rainbow trout protein coding transcripts obtained from Illumina sequencing (green bar) (A). Average length of UniGenes of model fish species and rainbow trout UniGenes that are available in the NCBI database (red bars) compared with the average length of rainbow trout protein coding transcripts obtained from Illumina sequencing (green bar) (B). The high number and short length of rainbow trout UniGenes suggest incomplete partial sequences. Illumina sequencing and Illumina/Trinity assembly resulted in 44,990 protein-coding transcripts with an average length of 1.97 kb, which is very close to number and average length of UniGenes in model fish species.

So far, the international effort of sequencing the rainbow trout transcriptome has led to the discovery of 136,979 UniGenes (NCBI UniGene downloaded August, 2014), 1,610 genes and 13,166 proteins that are available in the public NCBI database [10]. Coding sequences were annotated in a recent assembly of the rainbow trout genome [14], however, UniGene sequence information is not yet updated at NCBI. The number and average length of the rainbow trout protein coding transcripts identified in this study (44,990 transcripts; 1.97 kb) are similar to the number and average length of UniGenes from model fish species (Fig. 3). For example, zebra fish has 53,558 transcripts with a 1.04 kb average length. These data suggest that this sequencing project has captured the vast majority of the rainbow trout transcriptome. The protein coding Trinity transcripts are available at the USDA/NAGRP website http://www.animalgenome.org/repository/pub/MTSU2014.1218/

Grabherr *et*. *al*. found that Trinity was more sensitive than some other assemblers (Trans-ABySS, SOAP, Cufflinks and Scripture) in terms of percentage of full-length transcript reconstruction [57]. In another study comparing *de novo* assembly by various assemblers (SOAPdenovo, ABySS, Trans-ABySS, Oases and Trinity), Trinity assembly gave the highest (90%) RMBT value (Reads that can be mapped back to transcripts) and that the Trinity transcripts aligned better to the reference genome, indicating high quality of the transcripts [69]. One reason for the high quality of the transcripts constructed by Trinity may be its use of a fixed k-mer approach. In a previous study, Zhao *et*. *al*. found an increase in frequency of incorrect assemblies and artificially-fused transcripts by applying a multiple k-mer approach to the assemblers [69].

### Prediction of full-length cDNAs

Illumina sequencing in conjunction with Trinity assembly provided a platform for identification and characterization of full-length cDNAs without the need for laborious cloning/primer walking approaches. Putative gene identification was done first by BLASTx against the NR protein database and then by identification of coding regions using ESTScan. ESTScan uses a Markov model to recognize the bias in hexanucleotide usage that exists in coding regions compared to non-coding regions [58]. In the context of this work, whenever an ORF began and ended inside a contig it was considered as full-length cDNA. This means if the ORF began at the first base and ended at the last base, it was not considered as full length. A total of 15,736 putative full-length cDNAs with an average length of about 2.4 kb were identified. In addition, TransDecoder [http://transdecoder.sf.net] identified 25,705 unique transcripts with complete coding sequences. Full-length transcripts identified by the ESTScan and TransDecoder were aligned to the reference genome using BLAT [67]. There were 9,000 (57.2%) and 14,213 (55.3%) unique transcripts mapped at 90% of their total length, respectively. The average lengths of the full-length cDNAs were more than that of Atlantic salmon obtained from ESTs using TargetIdentifier (17,399 cDNAs with average length 1.36 kb). The same study reported 10,453 full-length cDNAs from the 51,199 rainbow trout ESTs [75]. A well-characterized full-length cDNA set from rainbow trout will be necessary for the annotation of the rainbow trout genome sequences as well as for comparative, structural and functional genomics studies.

### Assessment of the sequenced rainbow trout transcriptome

In order to assess the level to which the rainbow trout transcriptome has been captured, the 44,990 reference transcripts were BLASTx searched against reference proteome sets of seven different model fish species with known reference genomes. Out of 44,990 reference transcripts, a total of 30,880 (68.3%) sequences matched to protein sequences of all seven fish species and 37,753 sequences (83.9%) matched to protein sequences of at least one fish species with a cut off E value of 1.00E-10. These findings suggested a high degree of sequence conservation and homology with these fish species. Variable numbers of significant hits were identified within each species; *Danio rerio* (40.11%), *Oreochromis niloticus* (53.10%), *Takifugu rubripes* (34.73%), *Tetraodon nigroviridis* (50.24%), *Gadus morhua* (67.69%), *Gasterosteus aculeatus* (49.21%) and *Oryzias latipes* (48.14%) with cut off E values of 1.00E-10 (Table 3). Similar levels of homology to model fish species were reported in a catfish EST project (54% to 57%) [71] and a common carp transcriptome study (47.7% to 54.2%) [76]. To allow a fair comparison of the rainbow trout protein coverage with that expected between fish species with complete known reference genomes, cDNA sequences from each fish species were searched against complete reference proteome sets of other fish species using BLASTx search with a cut off E value of 1.00E-10. *Gadus morhua* cDNA sequences had hits to 64.97% (15,022 out of 23,118) proteins of *Tetraodon*, *Takifugu rubripes* sequences had hits to 64.45% (17,775 out of 27,576) proteins of *Gasterosteus aculeatus* and *Danio rerio* sequences had hits to 66.43% (17,779 out of 26,763) proteins of *Oreochromis niloticus* (data not shown). Since rainbow trout protein coverage observed in this study is within the expected range, we anticipate that the project has captured the vast majority of the rainbow trout transcriptome.

**Table 3:** Summary of BLASTx search analysis of rainbow trout sequences against different model fish species with known reference genomes.

	No of protein having hits to rainbow trout proteins	% of proteins with hits / total No of proteins in species
*Takifugu rubripes*	16,621	34.73% of 47,856
*Danio rerio*	16,345	40.11% of 40,747
*Oryzias latipes*	11,854	48.14% of 24,619
*Gasterosteus aculeatus*	13,409	49.21% of 27,248
*Tetraodon nigroviridis*	11,617	50.24% of 23,123
*Oreochromis niloticus*	14,206	53.10% of 26,753
*Gadus morhua*	14,961	67.69% of 22,100

### Functional annotation and gene ontology analyses

**Fig 4:**
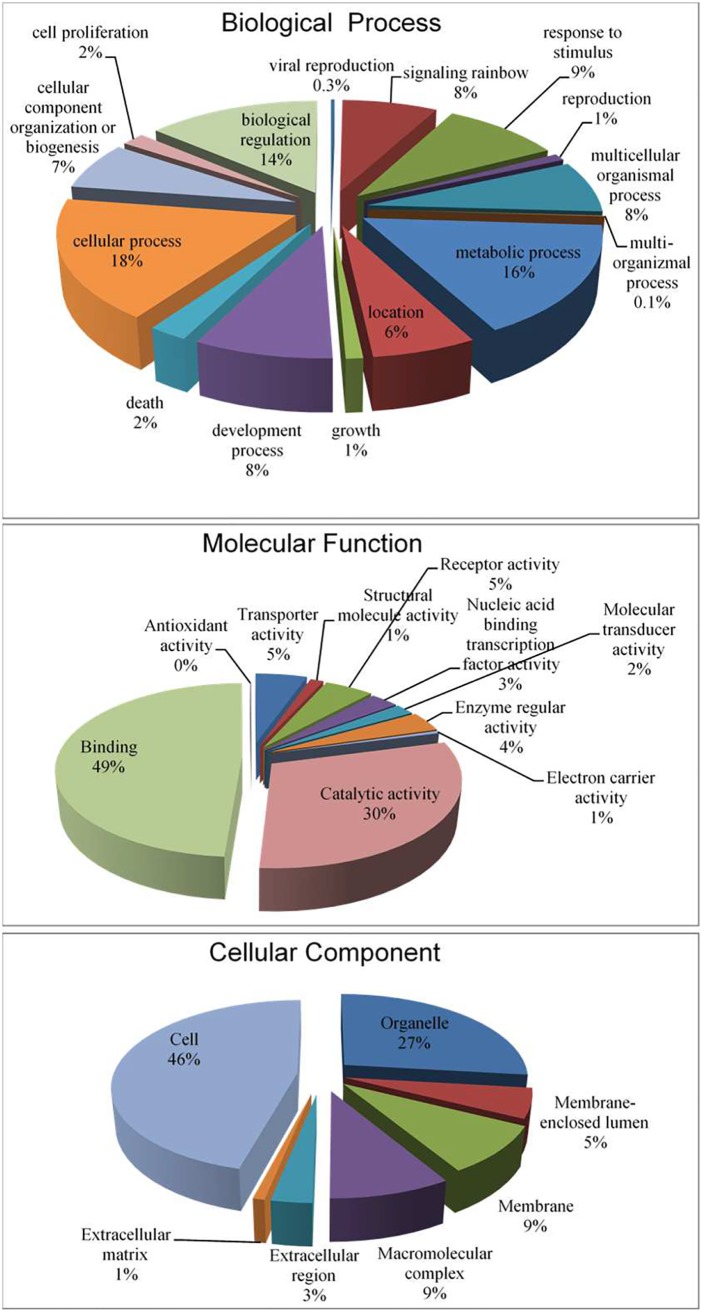
Gene Ontology (GO) assignment (2^nd^ level GO terms) of the rainbow trout of 13 lanes of Illumina Trinity assembly. Biological processes constitute the majority of GO assignment of transcripts (22,416 counts, 49%), followed by cellular components (12,793 counts, 28.1%) and molecular function (10,325 counts, 22.67%).

Gene ontology provides organized terms to describe characteristics of gene products in three independent categories: biological processes, molecular function, and cellular components [61,62]. Functional annotation of the Illumina/Trinity transcriptome contigs was performed by BLASTx search against the NCBI NR protein database using the Blast2GO suite [60]. The BLAST result findings were used to retrieve the associated gene names and Gene ontology (GO) terms in all three areas of ontologies. BLASTx results showed that biological processes constituted the majority of GO assignment of the transcripts (22,416 counts, 49%), followed by cellular components (12,793 counts, 28.1%), and molecular function (10,325 counts, 22.67%). The biological processes category showed that 18% of the rainbow trout genes were associated with cellular processes, 16% with metabolic processes, and 14% with biological regulation (Fig. 4). The molecular function category showed that 49% of the genes were associated with binding and 30% with catalytic activities. Of the cellular components, 46% of the rainbow trout genes were components of the cell and 27% were related to cellular organelles (Fig. 4).

Previously, we performed functional annotation of rainbow trout transcripts sequenced using Sanger based and 454-pyrosequencing techniques [29]. Compared to the Illumina/Trinity assembly, there were some noticeable differences in distribution of genes in all three areas of ontologies (data not shown). The most noticeable difference was observed in distribution of genes in biological process. As an example of the previous assembly, in the biological process category the highest number of transcripts were associated with biological regulation and cellular processes (25% each) followed by metabolic processes (18%). Similarly, in the molecular function category, a larger number of transcripts was found to be associated with binding function (46%) than with catalytic activity (32%). In the cellular component category, transcripts associated with the cell and organelles were 59% and 24%, respectively. Possible reasons for these differences may include variations in nature of cDNA libraries (non-normalized in this assembly versus normalized in the previous assembly) and number of sequences used to retrieve GO terms (161,818 versus 44,990). In addition, Illumina data have higher coverage and are expected to be more representative of the transcriptome. These dissimilarities may have resulted in differences in the number and types of genes captured by the sequencing projects, which might have resulted in slightly different GO distribution profiles.

### Taxonomic analysis

BLASTx top-hit species distribution of the gene annotations showed the highest number of matches to Nile tilapia (*Oreochromis niloticus*) followed by Zebrafish (*Danio rerio*) and Atlantic salmon (*Salmo salar*) (data not shown). Other fish species in the BLASTx top-hit list were Japanese puffer fish (*Takifugu rubripes*), puffer fish (*Tetraodon nigrovirdis*) and European sea bass (*Dicentrarchus labrax*). Most of the species on the top hit list were fishes, suggesting high quality of the assembled genes and a high level of phylogenetic conservation of genes between rainbow trout and other fish species.

**Fig 5:**
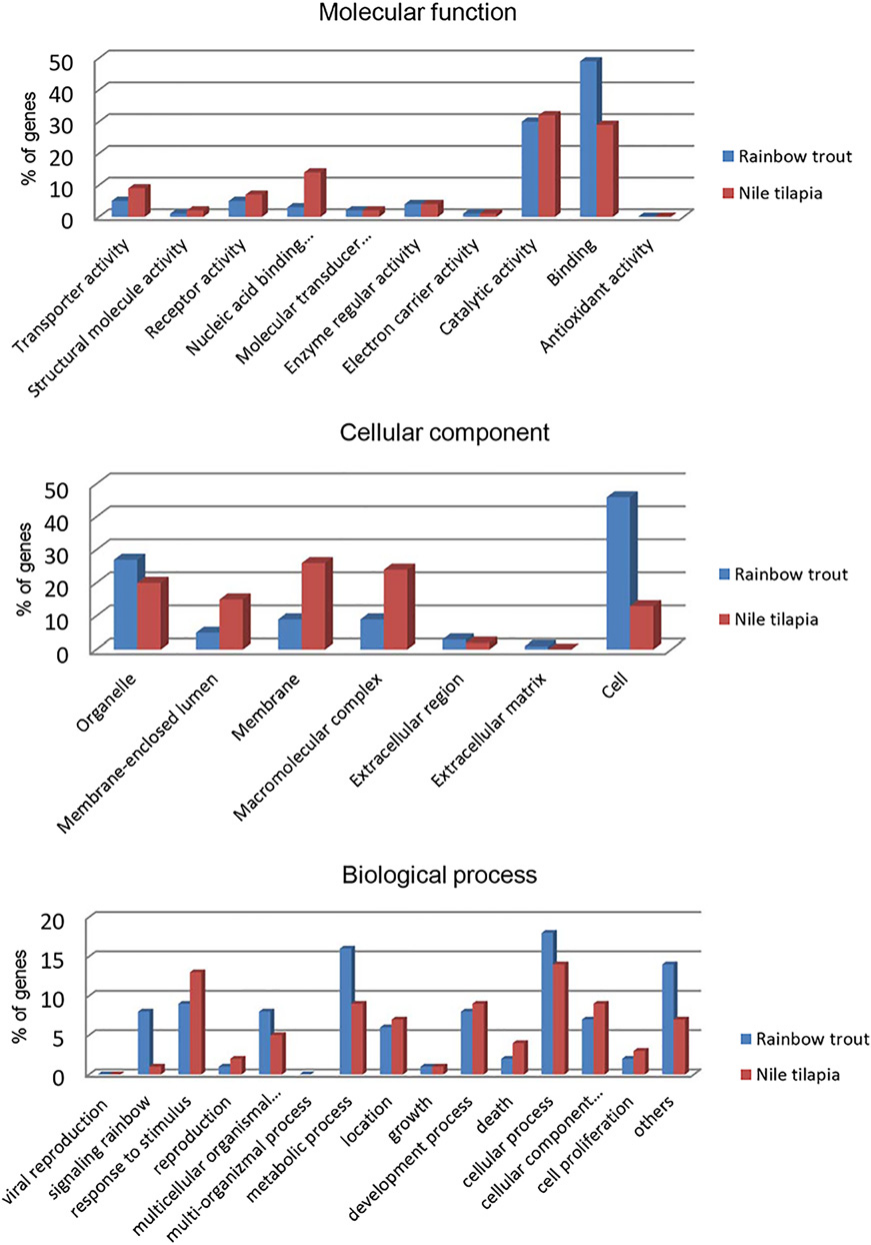
Gene Ontology (2^nd^ level GO terms) comparison of rainbow trout and Nile tilapia. GO comparison shows a high resemblance of GO terms between rainbow trout and Nile tilapia (Oreochromis niloticus).

As Nile tilapia showed high similarity to rainbow trout on the BLASTx top hit species distribution, the transcriptome of rainbow trout was compared to that of the Nile tilapia (Fig. 5). Gene ontology for biological process and molecular function showed a homogeneous distribution of GO terms of transcripts between rainbow trout and Nile tilapia, suggesting that our transcriptome from Illumina/Trinity assembly represents all transcribed genes of rainbow trout. However, there were some slight differences in GO distribution of transcripts, especially in the cellular component category (Fig. 5). This variation in GO distribution may be attributed to differences in the sequencing approaches used for rainbow trout and Nile tilapia as well as their phylogenetic differences.

### Characterization of housekeeping and tissue-specific genes

An important outcome of this transcriptome sequencing project was identification of housekeeping and tissue-specific genes from 13 vital tissues. By mapping reads from each tissue to the Illumina/Trinity transcriptome reference, we identified a total of 7,678 (17.0%) housekeeping transcripts expressed in all 13 tissues with a minimum of 1 RPKM value in each tissue (S1 Table). In comparison with mammals, a wide range of housekeeping gene percentages (1–38%) were reported in the mouse and human genomes using chip hybridization, MPSS (massive parallel signature sequencing) and next generation sequencing technologies [48,63,77]. Clearly, the differences are due to variations in technologies, number of tissues included, and nature of the duplicated rainbow trout genome.

**Fig 6:**
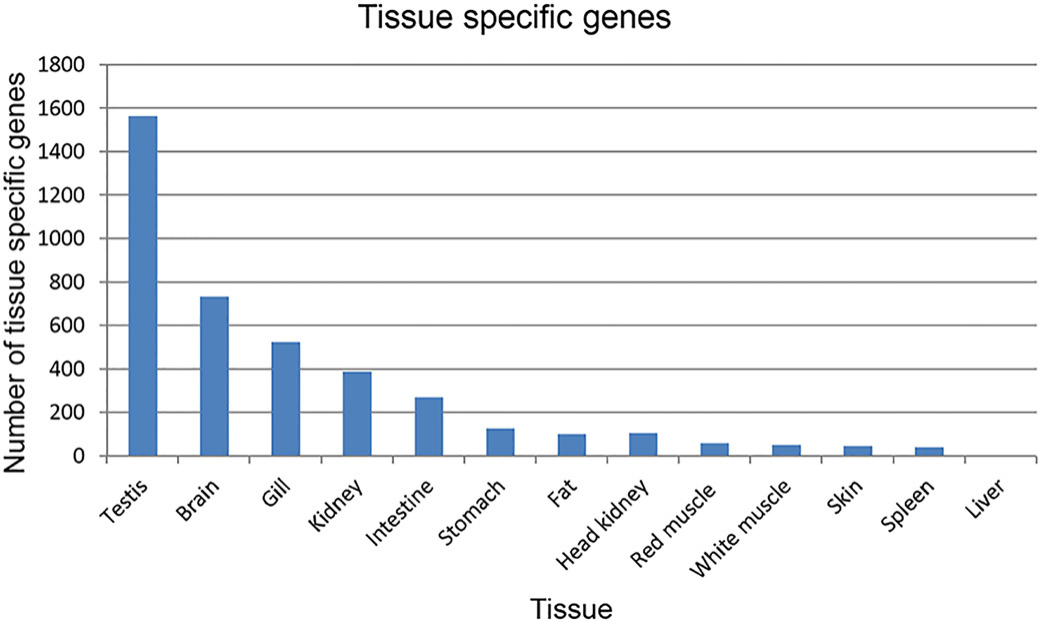
Number of tissue-specific genes predicted in different tissues. A transcript was classified as tissue-specific if it had an expression level in one tissue that is ≥ 8 fold higher in all other tissues.

Regarding the tissue-specific genes, a total of 4,021 transcripts with predominant expression in various tissues were identified in this dataset (Fig. 6). The level of gene expression of each of these tissue-specific genes was at least 8-fold higher in one tissue relative to the rest of the tissues. Using these criteria, there was no tissue-specific gene that matches any housekeeping gene in the dataset. Testis expressed the highest number of tissue-specific genes followed by brain, gill, and then kidney. Conversely, liver expressed the lowest number of tissue-specific genes followed by spleen, skin, and then white muscle (Fig. 6 and S2 Table). A similar trend of tissue specificity was observed in the human and mouse genomes [77]. Examples of the highly expressed genes shown in S2 Table include two brain transcripts that had expression levels more than 30 fold higher than the rest of the tissues. Of them, metabotropic glutamate receptor-5 is involved in signal transduction for glutamatergic neurotransmission in the human brain [78,79], and GABA (gamma-aminobutyric acid) receptor A is the principal inhibitory neurotransmitter in the mammalian central nervous system [80]. In skin, one of the three most highly expressed proteins is lily-type lectin which is a predominant protein in mucus of fish skin and provides important innate immunity [81,82]. Similarly, myosins and troponins were among the most highly expressed tissue-specific transcripts predicted in muscle, both of which play important roles in muscle contraction. In red muscle, four transcripts characteristic of slow (red) muscle were identified (Slow myosin light chain, Troponin-I, Slow skeletal muscle, Slow troponin-T family-like, and Slow myosin heavy chain-1). The tissue-specific expression results warrant further work to reveal how expression patterns are regulated in different tissues and how the functions of genes are influenced by the cellular context.

Gene ontology comparison of housekeeping and tissue-specific genes showed differences in patterns of GO distribution. For example, in the molecular function category, the percentage of transcripts involved in the transport, receptor activities, and DNA binding were notably higher among tissue-specific genes than housekeeping genes (3.8%, 3.0%, 1.4% versus 1.2%, 0.7%, 0.7%; respectively). Conversely, the percentage of transcripts involved in protein binding was greater among housekeeping genes in comparison to tissue-specific genes (26.2% versus 11.2%; respectively). More than half of the DNA binding transcripts have tissue specific expression, similar to the proportion reported in humans [77]. Additionally, in the cellular component category relatively more tissue-specific transcripts were associated with plasma membrane than transcripts from housekeeping genes (1.1% versus 0.7%; respectively). Conversely, more genes connected with the nucleus, cytoplasm and mitochondrion were classified as housekeeping genes (3.3%, 2.6%, 2.2% versus 2.3%, 1.6%, 0.6%; respectively). Further, in the biological function category, there were more tissue-specific genes linked to signaling, developmental processes, and response to stimulus (2.6%, 6.6%, 0.7% versus 1.7%, 4.6%, 0.3%; respectively). Similar trends in gene ontology comparisons between tissue-specific and housekeeping genes have been reported in mammals [77].

Taken together, these data indicate major biological role of the housekeeping genes in performing basic cellular functions needed to sustain life including metabolism, cellular processes, and biological regulation. However, tissue-specific genes were more involved in specialized functions such as signaling, responding to stimuli, development, organismal process, etc., suggesting diverse and specialized roles of tissue-specific genes in the cell.

### Complexity and composition of tissue-specific transcriptome

In an attempt to investigate the tissue complexity and composition of the rainbow trout transcriptome, the first question we asked was how many transcripts are expressed in a tissue? From 16,000–32,000 genes (at RPKM threshold of 0.5) were found to be expressed in the 13 studied tissues (Table 4). This range is slightly higher than what has been reported (12,170) in various mammalian tissues using RNA-Seq data at the same RPKM threshold [77]. The difference may be attributed to the duplicated nature of the rainbow trout genome. Other studies utilizing non-RNA-Seq experimental techniques reported expression of about 10,000–30,000 genes in different mammalian tissues [83–85]. Our data suggested that expression of about 35–71% of total genes (at RPKM of 0.5) seems to account for all basic and specialized functions of the 13 studied tissues (Table 4). This expression level is marginally different from the level reported in humans (61%-84%) using MPSS, but at less stringent conditions (RPKM threshold of 0.3) [63].

**Table 4:** Number of genes expressed in 13 rainbow trout tissues at different RPKM threshold.

Tissue	RPKM ≥5.0		RPKM≥ 1.0		RPKM≥ 0.5		RPKM ≥0.1	
	Number of genes expressed	Fraction of total genes	Number of genes expressed	Fraction of total genes	Number of genes expressed	Fraction of total genes	Number of genes expressed	Fraction of total genes
White muscle	2,949	0.06	10,798	0.24	15,970	0.35	27,593	0.61
Red muscle	6,425	0.14	18,991	0.42	24,136	0.54	33,079	0.74
Head kidney	7,461	0.17	19,699	0.44	24,368	0.54	32,022	0.71
Skin	6,646	0.15	20,951	0.47	27,796	0.62	38,669	0.86
Spleen	10,277	0.23	22,150	0.49	26,009	0.58	32,850	0.73
Fat	9,584	0.21	22,837	0.51	27,059	0.60	35,251	0.78
Testis	16,374	0.36	26,385	0.59	30,289	0.67	38,027	0.85
Kidney	12,253	0.27	25,856	0.57	29,964	0.67	36,783	0.82
Gill	13,804	0.31	26,149	0.58	29,757	0.66	36,440	0.81
Brain	11,464	0.25	27,151	0.60	32,053	0.71	39,697	0.88
Intestine	13,655	0.30	27,018	0.60	31,168	0.69	38,186	0.85
Liver	5,181	0.12	16,293	0.36	21,236	0.47	29,698	0.66
Stomach	6,982	0.16	19,462	0.43	24,460	0.54	33,807	0.75

**Fig 7:**
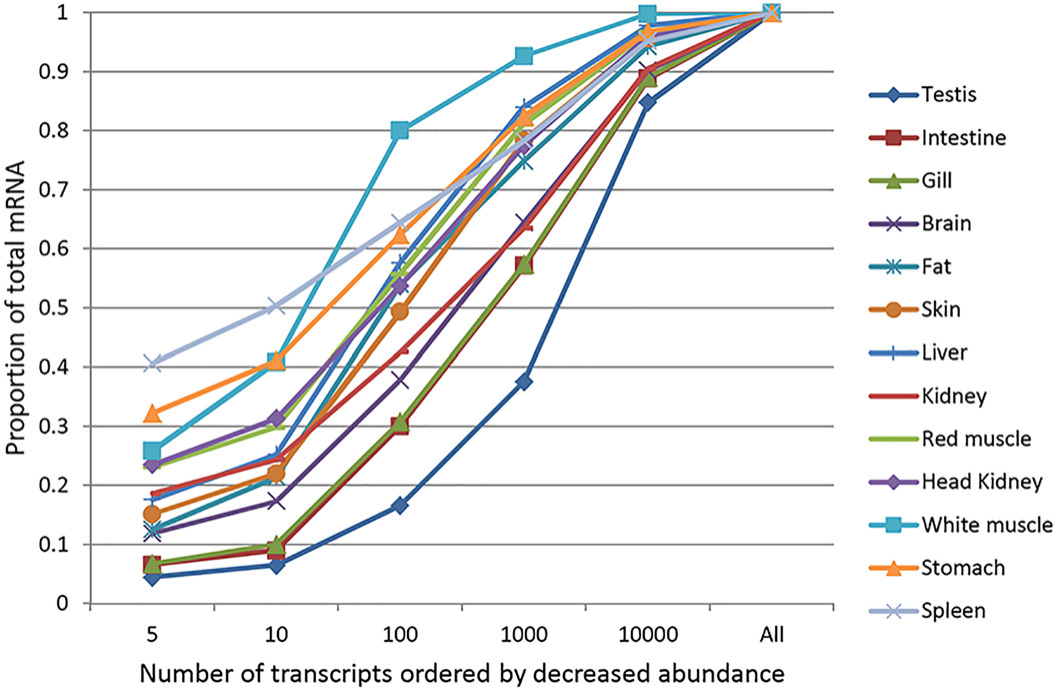
Distribution of gene abundance in various tissues. Proportion of the transcriptome contributed by the most abundant genes is plotted in various tissues. In testis, intestine, gill and brain, there was little contribution of the most highly expressed genes to the mRNA pool. Conversely, in white muscle, spleen, and stomach, a large fraction of the transcriptome was contributed primarily by the most highly expressed genes.

The second question we asked is how various tissues differ in composition and complexity of their transcriptomes? Brain, testis and intestine had complex transcriptomes in that they expressed larger percentages of the genes in the genome (Table 4) with a small fraction of the mRNA pool contributed by the most highly expressed genes (Fig. 7). On the other hand, white muscle and stomach had less complex transcriptomes, expressing fewer genes in the genome with a large fraction of the transcriptome contributed by the most highly expressed genes. As an example, the top hundred most highly expressed genes contributed 80% of the mRNA population in white muscle, while contributing only ~16% of the mRNA pool in testis (Fig. 7). Similar trends in transcriptome complexity were reported from previous studies in mammals [63,77] suggesting conservation of the tissue-specific expression patterns. Conserved expression of more than a third of the core tissue-specific gene expression was reported across major vertebrate lineages [86].

**Fig 8:**
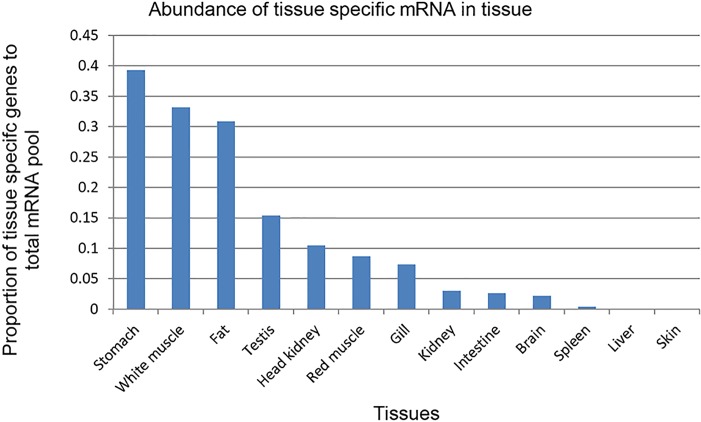
Transcript abundance of tissue-specific genes in various tissues. White muscle, stomach, and fat showed high abundances of tissue specific transcripts, while, skin and liver exhibited low abundance of tissue-specific transcripts.

The third question we asked is what is the contribution of the tissue-specific genes to the transcription pool in different tissues? Stomach, white muscle and fat had high abundances of tissue-specific transcripts; and skin, liver, spleen, brain, kidney and intestine had low abundances of tissue-specific transcripts (Fig. 8). Although stomach, white muscle, and fat expressed relatively fewer tissue-specific genes (51–127 genes), these transcripts significantly contributed to the total cellular mRNA pool (31–39% of total mRNA) (Fig. 8 and S2 Table). Conversely, in brain, kidney, and intestine, which expressed a large number of tissue-specific genes (734, 390 and 271 genes, respectively), these genes contributed only 2–3% of total cellular mRNA. These results indicate wide variation in the number of genes and regulation of gene expression that determine tissue specificity.

This complexity in the expression pattern of genes may be explained in terms of not only the degree of specialization but also the types of cells in each tissue. For example, brain has a variety of cells specialized for equally important but different functions. As different cell types express different cell-specific genes, tissue as a whole has a large collection of equally important tissue-specific genes expressed at comparable rates (Fig. 8). In contrast, in fat, a majority of gene expression is directed to the manufacture of necessary enzymes to carry out basic fat metabolic pathways. Therefore, there is an abundance of a relatively small number of fat metabolic transcripts. The other possibility is that most of the cells in fat tissues are alike and the genes taking part in some important function may be expressed highly in all cells so that their mRNA population may be dominated in non-normalized libraries.

A multivariate Principal Component Analysis (PCA) analysis was applied to cluster tissues types according to gene expression patterns. Two dimensional covariance matrix of the different tissue samples revealed distinct expression of both the spleen and the kidney (S1 Fig.). Recently, we reported a detailed expression in the spleen transcriptome in rainbow trout [87]. The distribution of rest of the tissues were clearly classified into 2 clusters (head kidney, red muscle and stomach) and (testis, gill, fat, skin, intestine, brain, white muscle and liver).

### Comparison of the Trinity assembly to the reference genome annotation

Berthelot et al used a gene models approach based on both a genome and a transcriptome sequences to predict 46,585 annotated protein-coding genes [14]. To assess the *de novo* transcriptome assembly approach used in this study against the gene models approach used by Bethelot et al, we first ran a reciprocal BLAST search between the two datasets. A total of 4,146 contigs of the Trinity assembly (9.2%) including, 710 full-length sequences, did not match any mRNA sequences identified in the genome reference (BLASTn, E value > 1.00E-10). These contigs may represent unannotated, incomplete, or absent loci in the trout genome. On the other hand, 2,641 mRNAs sequences in the genome reference did not match any of the Trinity contigs. All teleost protein sequences were used, at least partially, to annotate the trout genome [14]. Therefore, some of these 2,641 missing transcripts may represent predicted gene models that are not expressed in rainbow trout, at least in the single individual used in this study.

In addition, we ran BLASTx of the two datasets against the zebrafish proteome (with a cut off E value of 1.00E-3, downloaded from Ensembl 11/17/2014). A total of 19,390 (44.9%%) of the zebrafish proteins had hits by at least one of the Trinity contigs, compared to 21,119 (48.9%) proteins in case of the trout genome mRNA sequences. There were 16,046 (39.6%) zebrafish protein hits shared between the two datasets. A total of 4,378 and 1,077 transcripts of the Trinity and the genome reference mRNAs had no hits to the zebrafish proteome, respectively. When the two datasets were compared by BLAST with proteome sequences of seven model fish species (with known genomes), there were 3,297 and 195 transcripts of the Trinity and the trout genome reference mRNAs with no hits, respectively. TransDecoder recognized 25,705 (57.1%) and 38,313 (82.2%) transcripts with complete ORFs in the Trinity and the trout genome mRNAs, respectively. Taken together, the comparison of *de novo* transcriptome assembly approach (used in this study) and the gene models approach used by Bethelot et al, indicate some differences in the transcripts/annotations identified by each method. It is worth mentioning that, in this study, the transcriptome was sequenced from the Swanson clonal line which is the same source used for the rainbow trout genome sequencing. However, a large proportion of the transcriptomic data used by Berthelot and coworkers to annotate the genome came from a different clonal line [14].

To assess the percentage of the mappable Trinity transcripts to the genome reference, Trinity transcripts were aligned to the reference genome using BLAT and then the best hits were selected using the pslReps program of the BLAT suite [67]. BLAT hits were classified according to the percentage of Trinity sequence identity covering the reference coding sequence of the genome. There were 1,434 (3.2%); 25,860 (57.5%) and 38,367 (85.3%), unique Trinity transcripts mapped at 100%, 90% and 50% of coverage, respectively. These results, at least partially, validate the Trinity assembly. However, the current version of the genome sequence is still not complete which prohibits a complete assessment of the Trinity assembly based on the BLAT results.

In an effort to find novel loci (not annotated) in the genome, sequence reads were mapped to the genome reference using TopHat and Cufflinks software packages [64]. A total of 223,751 gene loci were predicted with 286,561 potential transcripts (average of 1.28 transcripts/gene). These gene loci were filtered against the trout genome annotated loci first by BLASTn against the mRNAs (E value 10^-5^) and then by comparing the genome annotation coordinates (gtf files) using an in-house script (available upon request). Using this approach a total of 78,592 novel loci were identified. Further investigation used TargetIdentifier [66] and TransDecoder [http://transdecoder.sf.net] to determine novel genes with ORFs. TargetIdentifier recognized 10,195 full ORFs and TransDecoder identified 12,652 ORFs with 3,420 complete ORFs. There were 1,432 transcripts, with complete ORF common between the TargetIdentifier and TransDecoder datasets. Using an in-house script based on a BLASTx to the NR database with and E value 10–3, there were 128 genes with 100% matches and 832 genes with 80% matches to the NR database not annotated in the reference genome. After redundant removal, 11,843 transcripts were recognized as new transcription loci. To provide a comprehensive list of all new transcripts that were identified in this study (not annotated in the trout genome), those 11,843 were screened to remove redundancy with the 4,146 contigs of the Trinity contigs that had no match with any mRNA sequences in the genome reference. A total of 14,827 (11,843+2,984) were counted as new transcripts. FASTA and annotation (gtf) files of those new transcripts are provided (S1 and S2 Datasets) and available for download http://www.animalgenome.org/repository/pub/MTSU2014.1218/

### Comparison of the Trinity assembly to the marine rainbow trout transcriptome

The anadromous steelhead (*Oncorhynchus mykiss*) transcriptome was recently sequenced [31]. To assess gene expression associated with adaptation to ecological and environmental factors in the marine versus the freshwater rainbow trout, we ran a reciprocal BLASTn search. A total of 8,312 contigs of the Trinity assembly (18.4%) did not match any sequences in the marine rainbow trout (BLASTn, E value > 1.00E-3). On the other hand, 12,207 (9.3%) marine rainbow trout transcripts did not match any of the Trinity contigs. These results should be considered with caution because of the unbalanced amount of data (~1.167 billion paired-end reads [100bp] in the freshwater trout, compared to 41 million 76-mer reads in in the marine trout). Gene ontology comparison of the marine versus freshwater unmatched transcripts did not show significant gene enrichment for salinity adaptation (data not shown).

### Assessment of alternative transcription/splicing

Trinity assembler is capable of predicting alternative splicing events. There were a total of 287,593 Trinity contigs longer than 500 nucleotides that had hits to the NR protein database. A total of 92.5% (266,188) of these contigs were part of the components with more than one contig, indicating the contigs had alternative transcription/splicing. However, these contigs may also be separately expressed from paralogous genes. Therefore, the TopHat and Cufflinks read mapping to the genome, described above, were used to assess the percentage of alternative transcription/splicing events. Out of 223,751 predicted genes, 27,471 (12.8.) genes had at least two transcripts from alternative transcription/splicing; 4,663 (2.08%) genes had five and more transcripts and 634 genes had 10 or more transcripts. A total of 1,064,892 exons were detected yielding an average of 4.75 exons/locus.

The low percentage of genes with alternative splicing is unexpected because alternative splicing is one of the important components adding functional complexity to vertebrates; in humans about half of the genes have at least one splice variant [88]. However, because of the whole genome duplication event in teleost fish, many genes have paralogous duplicates [89–91]. Indeed, gene duplication can lead to loss of alternative splicing of genes [92,93] and many of the splice variants present in an ancestor are found to be expressed separately from duplicated genes in teleost fish [94]. The rate of alternative splicing was lowest (17%) in the highly duplicated genome of zebrafish compared to the compact genome of the pufferfish (43%) [95]. Availability of a complete and annotated sequence of the rainbow trout genome is needed to fully characterize transcripts representing splice variants and separately expressed sequences of paralogous genes.

## Conclusion

High throughput Illumina sequencing of non-normalized cDNA libraries from 13 tissues was used together with the Trinity assembler to generate a high-quality draft of the rainbow trout transcriptome. A single doubled haploid rainbow trout fish, from the same source used for the rainbow trout genome sequence, was used to address problems associated with the nature of the rainbow trout duplicated genome. Results of the *de novo* approach, used in this study, were compared to results of the gene models approach that was used in annotating the genome sequence. A total of 14,827 sequences were identified as new transcripts (not annotated in the trout genome). A digital gene expression atlas revealed 7,678 housekeeping and 4,021 tissue-specific genes. In addition, expression of 16,000–32,000 genes (35%-71% of the transcriptome) was revealed in various tissues. White muscle and stomach showed the least complex transcriptomes, with high fractions of their total mRNA expressed by a small number of genes. In contrast, Brain, testis and intestine had complex transcriptomes with large numbers of genes involved in their gene expression.

## Supporting Information

S1 Table List of tissue specific transcripts and their expression in various tissues. (XLSX)

S2 Table List of housekeeping transcripts and their expression in various tissues. (XLSX)

S1 Dataset FASTA file of the newly (not annotated in the genome) identified transcripts. (FA)

S2 Dataset Genome annotation information (Gene transfer; FTF) file of the newly (not annotated in the genome) identified transcripts. (GTF)

S1 Fig Principal Component Analysis (PCA) analysis showing clusters of tissues types according to gene expression patterns. (TIF)
