# Supplementary figures and images for: Transcriptome Assembly, Gene Annotation and Tissue Gene Expression Atlas of the Rainbow Trout
Source: PLoS One. 2015 Mar 20;10(3):e0121778. doi: 10.1371/journal.pone.0121778 (PMC4368115; doi:10.1371/journal.pone.0121778)

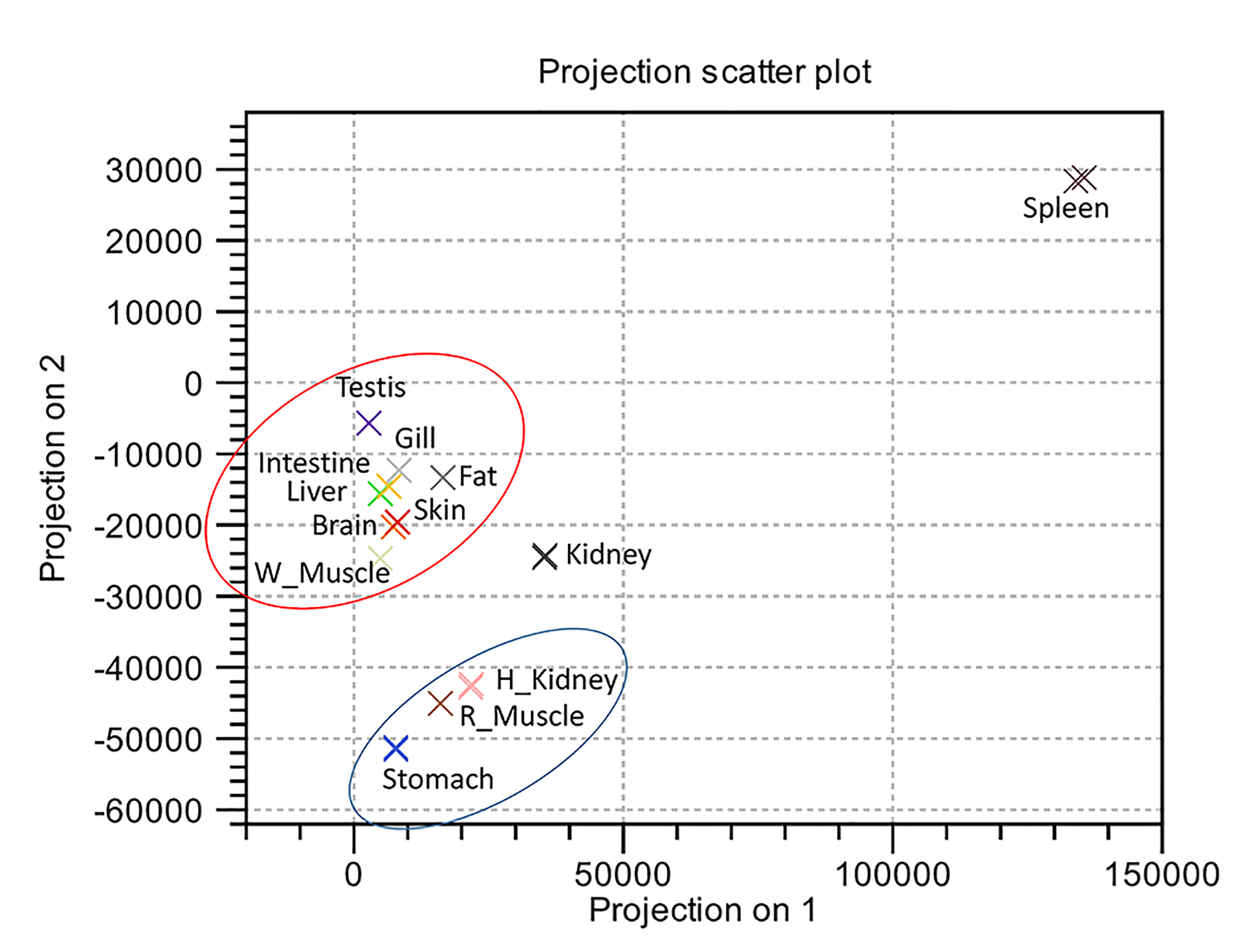

Supplement: S1 Fig — (TIF) [file pone.0121778.s005.tif]
